# What College Students Post About Depression on Facebook and the Support They Perceive: Content Analysis

**DOI:** 10.2196/13650

**Published:** 2020-07-17

**Authors:** Scottye Cash, Laura Marie Schwab-Reese, Erin Zipfel, Megan Wilt, Megan Moreno

**Affiliations:** 1 College of Social Work The Ohio State University Columbus, OH United States; 2 Department of Health & Kinesiology Purdue University West Lafayette, IN United States; 3 Department of Pediatrics University of Wisconsin-Madison Madison, WI United States

**Keywords:** social media, depression, college students, qualitative

## Abstract

**Background:**

College students frequently use social media sites to connect with friends. Increasingly, research suggests college students and other young adults seek mental health-related support on social media, which may present a unique venue for intervention.

**Objective:**

The purpose of this study was to examine college students’ perceptions about displaying feelings of depression on Facebook and, in turn, how their social media friends responded.

**Methods:**

A primarily quantitative online survey with open response questions was distributed to students at four US universities. Qualitative responses were analyzed using content analysis.

**Results:**

A total of 34 students provided qualitative responses for analysis, these students were 85.3% female, mean age 20.2 (SD=1.4) and 20.6% racial/ethnic minority. Students who reported posting about depression often expressed an emotion or feeling but did not use the word “depression” in the post. Approximately 20% posted language about a bad day, and 15% posted a song or music video. Only one person reported posting a statement that directly asked for help. When friends responded to the posts, students generally perceived the responses as supportive or motivating gestures. Nearly 15% of friends contacted the individual outside of Facebook. One individual received a negative response and no responses suggested that the individual seek help.

**Conclusions:**

This study found that college students who post about depression often do so without directly referencing depression and that friends were generally supportive. However, no participants reported their social network suggested they seek help, which may suggest increasing mental health literacy, for both support seekers and responders, would be an opportunity to improve online mental health-related support.

## Introduction

Depression is a common health issue among college students [1-4]. Untreated depression may cause a number of adverse outcomes, including substance use, suicidal behaviors, and other psychiatric conditions [5-10]. Obtaining help with depression can be complicated by students’ inability to understand their symptoms, denial of symptoms, unwillingness to reach out for formal help, a lack of available mental health resources, and other complications associated with depression severity [2,3,11]. Innovative approaches are needed to increase the knowledge about depression and help-seeking resources while also reducing the stigma associated with mental health issues.

Internet-based or online social networks may be an important avenue for young people to seek and receive support related to mental health and to access mental health resources. In 2018, nearly all young people in the United States used the internet through home broadband or smartphones [12]. Social network site use is nearly as ubiquitous. In 2018, YouTube was used by the greatest percentage of 18- to 24-year-old Americans (94%), followed by Facebook (80%), Snapchat (78%), and Instagram (71%) [13]. Although social media use has been shown to be associated with increased mental health issues under some circumstances [14-18], a growing body of literature has shown that college students use social network sites, such as Facebook and Twitter, to discuss mental health concerns and seek support [19-22]. Moreno and colleagues [20] found that 25% of respondents had some evidence of depression symptoms in their Facebook posts, and 2.5% posted about symptoms that met the criteria for a major depressive episode. The authors also found that individuals who posted about depression were online more often than those who did not [20]. Support-seeking behaviors may be driving this increased use. One study demonstrated that young adults with a mental health diagnosis were more likely to seek support online and to engage in various other social connection-seeking activities than peers without a mental health diagnosis [21]. However, social media engagement may not be entirely positive. In another study of social media use among adolescents with depression diagnosis, participants used social media in ways that were likely to positively (eg, entertainment, humor) and negatively (eg, sharing risky behaviors, cyberbullying) impact their symptoms [22].

Few studies have examined the type of response that students receive from peers when they post about depression symptoms on Facebook. In one study, a positive association was found between depression symptom posts and peer responses, and peer responses were generally positive and supportive [20]. However, in another study, participants perceived depression symptom posts as “drama” and were reluctant to offer help or support [23]. This discrepancy may be due to differences in how individuals with depression perceive social interactions compared with nondepressed peers. One small study found that individuals who posted about depression *thought* they received less support, but a review of actual social support transactions found that posts about depression symptoms were associated with increased social support from Facebook connections [24].

Despite growing evidence about the objective use of Facebook and other social network sites to share emotions/experiences related to depression, relatively less is known about how young people perceive those experiences [25]. The purpose of this study was to determine how college students *perceived* their help-seeking posts about depression and the types of responses they received.

## Methods

### Design

Data for this analysis were extracted from a comprehensive mixed method, multisite, cross-sectional study conducted between September and November 2012. The purpose of the overall study was to assess multiple health outcomes among a college student population, including mental health and technology-related behaviors. Data were collected via an online survey with college students from four universities in the United States. The four universities were selected to provide geographic variation and a mix of public and private schools. Of these, three were in the Midwest and one was in the West. All participants in the overall study were invited to respond to all relevant questions, including the qualitative questions reported in this paper. As the Patient Health Questionnaire-9 (PHQ-9) includes questions regarding suicidal thoughts over the past 2 weeks, students were provided a list of Web-based mental health resources as a condition of the institutional review board (IRB) approval. This study was approved by each university’s affiliated IRB.

### Procedures

Paper fliers with a link to the online Catalyst WebQ survey (https://itconnect.uw.edu/learn/tools/catalyst-web-tools/webq/) were distributed to 662 college students taking biology, communications, psychology, and nursing classes during the Autumn semester in 2012. These specific classes were selected to be able to reach a diverse set of students, as many were introductory classes required across different majors in order to be able to reach a diverse set of students. Each college class that was included in the study had a general Listserv that the professors used to communicate with students. In all, two to three follow-up emails were sent via the course Listserv to all 662 students who received the flier. Students who accessed the survey were required to complete an informed consent. A US $5 Starbucks gift card was given to those who completed the survey. In one class, students who completed the survey received extra credit in addition to the gift card. To complete the survey, students were required to be between the age of 18 and 23 years, and this inclusion criterion was included in recruitment materials. Any respondents outside this age range were excluded from this analysis. The response rate for the overall survey was 43.3% (n=287). The final sample size for this study was 33 students, which represented 11% of the total sample.

### Sample

The sample for this study included all participants (n=33) who provided qualitative responses to the survey question on the nature of their and their friends’ responses to their Facebook posts and who had completed the questions on the PHQ-9.

A *t* test was used to determine if there were demographic and mental health differences between this study sample (n=33) and the sample of students who did not complete the open-ended questions (n=253). There was a statistically significant difference (*P*=.001) in the mean score on PHQ-9 between those who completed the qualitative Facebook questions (mean 9.06, SD 6.43) and those who did not complete those questions (mean 4.92, SD 4.25). No other statistically significant differences were identified between the full sample and the subset of participants in this study.

### Measures

Demographic data were collected from each participant, including age, race/ethnicity, relationship status, sexual orientation, and year in school.

#### Depression

Depression was measured using the PHQ-9. The PHQ-9 score was based on the instrument guidelines with the following categorization: <4=minimal depression, 5-9=mild depression, 10-14=moderate depression, 15-19=moderately severe depression, and 20-27=severe depression. A total score of 10 or more points was used in this study to indicate at least moderate depression systems, which was consistent with the prior use of the questionnaire [26]. The PHQ-9 was chosen to measure depression in this study because it has been validated in both adolescent and adult populations [27,28].

#### Qualitative Facebook questions

Participants were asked to identify if they had ever reached out on Facebook when feeling depressed and if yes, to describe the post(s). Participants were then asked to describe the nature of comments their friends made in response to a post, where they relayed some variation of feelings of depression. A copy of the questions are presented in Multimedia Appendix 1. The qualitative Facebook questions were developed by a study investigator, who based these questions on an internet survey about support-seeking behaviors used by adolescents when they were depressed [29]. Other researchers in the team then conducted an initial review of the Facebook questions for face validity and assessed them for question clarity. Prior to implementing the survey, the questions were also vetted by several college students.

### Analyses

The first purpose of the study was to identify themes in participant perceptions regarding depression-related Facebook posts and their friend’s responses. Classic content analysis [30-32] was used to examine responses to the two open-ended questions. Three investigators defined categories a priori (see Table 1). Investigators then participated in three rounds of practice coding. Discrepancies were discussed after each round of coding. An interrater agreement of 0.79 was obtained on a subset of 10 quotes before coding of the full subsample. The remaining data were divided among three coders and an open coding approach was used to categorize the total sample of open-ended responses. As ambiguous or unclear cases emerged, they were discussed and assigned the category agreed up on by all three coders. The final interrater agreement on all 33 statements was 0.90.

**Table 1:** Qualitative themes and definitions for participant posts and their friends’ responses.

Themes		Definition
**Participant post**		
	Emotion	Emotion or feeling that does not use the word depressed.
	Bad day	Reference to having a bad day or a bad one-time experience doing something.
	Song	Lyrics of a song or mention of a music video.
	Private message	A type of message that is only available to the recipient via Facebook, email, phone, text.
	Depressed mood	Reference to symptoms of a major depressive episode, which included (1) depressed mood, (2) decreased interest or pleasure in day-to-day activities, (3) increase/decrease in appetite, (4) increased/decreased sleep, (5) psychomotor agitation or retardation, (6) loss of energy, (7) feelings of guilt, worthlessness, negative self-appraisal, (8) indecisiveness, (9) difficulty in concentrating, (10) recurrent thoughts of death or suicidal ideation.
	Emoji/emoticon	The emoticon of a sad face.
	Quote	The use of someone other than the profile owner’s words to express emotions.
	Joke	Making fun of depression or being sarcastic.
	Asked for help	Follow-up the content of the post with saying need help.
**Friend response**		
	Support or motivating gestures	An individual saying supporting or motivating words to the participant.
	Asked a question	Questioned individual on how they were doing.
	Contact in alternative communication	An individual contacted the participant using some other form of communication, such as email, text, phone call, etc.
	Liked	An individual clicked like to their post or commented on Facebook.
	Private message	Contact the individual through nonpublic methods.
	Empathize	Understand and feel the feelings of another, “walk a mile in their shoes.”
	Sympathize	Compassion or commiserating with another, “I’m sorry that you are feeling this way.”
	Negative	An individual responding in an unsupportive way.
	No response	Received no response from friends after they posted.

We conducted bivariate analyses to assess if the theme of friend’s response differed by the theme(s) of the participant’s post. We also conducted bivariate analyses to assess differences between themes of the participant’s posts, by depression severity. To test for statistical significance with these analyses, while accounting for the small sample size and cell counts, we used Fisher exact test.

## Results

The mean age of the 33 participants was 20.2 (SD 1.4) years. The majority of participants were female (n=28, 85%) and Caucasian (n=26, 79%). Participants were at different stages in their studies, with 24% freshman (n=8), 18% sophomore (n=6), 36% junior (n=12), and 24% senior (n=8). Almost half of the participants were single (n=16, 48.5%), and only one person identified himself as homosexual.

The mean score on the PHQ-9 was 9.06 (SD 6.43; n=33). Based on the normed scoring criteria, those who scored 10 or more points were considered to be clinically depressed. Of note, 12% had minimal depression (n=5), 33% had mild depression (n=11), 27% had moderate depression (n=9), 6% had moderate to severe depression (n=2), and 12% had severe depression (n=4); three respondents had a score of 0 on the PHQ-9. Question 9 on the PHQ-9 assesses suicidal thoughts in the last 2 weeks. Of these, 33% indicated suicidal thoughts on several days (n=11) and 6%, nearly every day (n=2).

We used a multiple-response analysis, where participants could identify multiple ways in which they had posted about their depression on Facebook. Of these, 24% (n=8) most commonly reported that they posted their posting about their emotions, and 18% (n=6) referenced a bad day (Table 2). A total of four participants (12%) referenced a specific form of the word depression or an associated symptom. Of the participants, 15% (n=5) referenced to lyrics of a song or a music video and 6% (n=2) included quote from a song. Of these, 12% (n=4) reported that their friends responded via alternative communication approaches or through a private message; 6% (n=2) included an emoji/emoticon. Only 1% participant reported directly seeking help.

**Table 2:** Number and percentage of themes present in participant’s posts

Theme	n (%)^a^	Example quote
Emotion	8 (24)	“I’m tired of being treated like crap, etc.” “I just said I felt so alone.”
Bad day	6 (18)	“I made a couple of posts over the course of the past year. In one I posted something similar to ‘Oh man, something’s gotta give.’ Another one I remember saying ‘I’m calling all you angels.’” “Terrible day. Things couldn’t get any worse.”
Song	5 (15)	“Something with sad music, or a comment on how frustrated or hard life can be.” “I posted a music video that correlated with my mood that day.”
Private message	4 (12)	“I didn’t post something about it, I private messaged a friend for some advice.” “I did not post publicly. I talked to my best friend via Facebook messaging. I talked to her about my problems and what I should do.”
Depressed mood	4 (12)	“Depressed sounding post, which led to friends helping.” “Just a sad status.”
Emoji/emoticon	2 (6)	“Generic sad faces or ‘screw this’ posts.” “Sad face.”
Quote	2 (6)	“Just posted a quote about being stressed.”
Joke	1 (3)	“I have posted jokingly about particular days being emotionally difficult.”
Asked for help	1 (3)	“If anyone could help me get through a problem.”
Other	2 (6)	“It’s probably one of the hardest things when you wanna help someone but you know you can’t. And you know you should just worry about yourself but you just can’t help it.”

^a^Qualitative responses may have included multiple themes, so the column does not add up to 100%.

The most common perceived response to the participant’s Facebook depression post was gestures of support or motivation (n=13, 39%; Table 3). Almost 15% of the participants (n=4) mentioned that their friends responded via alternative communication approaches or through a private message. A total of four participants (12%) discussed receiving “likes.” One described a negative response to the depression-related display. No participants reported that their friends encouraged them to seek help.

**Table 3:** Number and percentage of themes present in friends’ responses.

Theme	n (%)^a^	Example quote
Support or motivating gestures	13 (39)	“All positive, sweet, telling me I will get through anything.” “All my close friends were there to encourage me and letting me know that everything will be okay.”
Asked a question	7 (21)	“If they are generic posts friends generally have to ask what is wrong. It is hard to tell who cares or who’s curious this way though.” “They ask what’s wrong or text, or message me to talk.”
Contact in alternative communication	4 (12)	“My best friend called me when she got the message and talked to me for a long time.” “She called me immediately.”
Liked	4 (12)	“A few people ‘liked’ it.” “People ‘liked’ the music video.”
Private message	4 (12)	“They make plans to hang with me and take my mind off of it.”
Empathize	2 (6)	“They would respond most often by agreeing with me and saying that they were having a hard time as well.”
Sympathize	1 (3)	“Tried to make me feel better, ‘hang in there,’ etc.”
Negative	1 (3)	“Negatively”
No response	1 (3)	“Usually they don’t respond...”

^a^Qualitative responses may have included multiple themes, so percentages do not add up to 100%.

We also explored the relationship between individuals’ Facebook post and their friends’ perceived response. When a person posted about having a “bad day,” 83% of his/her friends responded with support or motivation and 17% responded with sympathy. When he/she posted a song, song lyrics, music video, 40% received a response coded as support or motivation, and 60% “liked” the post. When the person made an emotional post, 62% received a support/motivation response, 13% were asked to communicate outside of Facebook, and 25% asked a question. When the person wrote that he/she had some depression or depression symptoms, half of his/her friends responded with a negative message and the other half used a private message to communicate. Finally, if the person made a joke about being depressed, he/she received an empathetic response. There was a significant relationship (*P*<.001) between how a person reached out on Facebook and his/her friend’s perceived response. We also explored the relationship between the PHQ-9 category and the type of post the person made on Facebook (Table 4). The themes of the participant posts varied by depression severity.

**Table 4:** Patient Health Questionnaire-9 categories by theme of participant post.

Depression severity	Song, n (%)	Private message, n (%)	Depressed mood, n (%)	Help, n (%)	Emoji, n (%)	Joke, n (%)	Bad day, n (%)	Emotion, n (%)	Quote, n (%)	Other, n (%)
Minimal (n=5)	0 (0)	1 (20)	1 (20)	1 (20)	0 (0)	0 (0)	1 (20)	1 (20)	0 (0)	0 (0)
Mild (n=11)	1 (9)	2 (18)	1 (9)	0 (0)	1 (9)	1 (9)	1 (9)	2 (18)	1 (9)	1 (9)
Moderate (n=9)	1 (11)	0 (0)	1 (11)	0 (0)	1 (11)	0 (0)	1 (11)	44 (44)	0 (0)	1 (11)
Moderate to severe (n=2)	1 (50)	0 (0)	0 (0)	0 (0)	0 (0)	0 (0)	1 (50)	0 (0)	0 (0)	0 (0)
Severe (n=4)	0 (0)	1 (25)	0 (0)	0 (0)	0 (0)	0 (0)	2 (50)	0 (0)	1 (25)	0 (0)

## Discussion

### Overview

The purpose of this study was to determine how college students *perceived* their help-seeking posts about depression and the types of responses received. In our sample, no participants reported using specific indicators of depression symptoms, as defined in the Diagnostic and Statistical Manual of Mental Disorders, Fourth Edition (DSM-IV; eg, guilt/worthlessness, hopeless, depressed mood) [33], which is contrary to the findings of two studies on Facebook posts by depressed individuals that found that some individuals used a language consistent with diagnosable depression symptoms [20,34]. Since our study focused on how individuals perceived their posts, this difference between our studies and the other studies suggests that participants in our study had altered perceptions of the content of and responses to their posts, which is consistent with some prior research on online support seeking [24]. In one prior study, more severe depression symptoms were associated with significantly reduced perceptions of Facebook-related support, despite increased levels of support found in the review of the text of the interactions [24]. A larger body of research has demonstrated a negative bias in how individuals with depression perceive social support and other social perceptions in offline interactions [35-37]. Alternatively, social desirability bias may account for the difference between participants’ perceptions and review of social media posts in prior studies [20,24,38]. Students may not report using specific depression symptoms due to concerns about social stigma. However, it is unclear why participants in this study would report posting about reaching out on Facebook regarding depression and then withhold that they used depression-specific language.

In addition to triangulating prior studies of Facebook-related mental health support, our study has several implications. In 2017, Facebook implemented a new mechanism for concerned friends and family to provide support for individuals who may be experiencing suicidal thoughts [39]. After a report is made, Facebook will send a message indicating someone expressed concern and will provide a list of resources (eg, suicide and crisis hotlines). Facebook also changed the “Like” function, so that individuals can indicate their reactions (eg, “Like,” “Happy,” “Wow,” “Sad,” and “Angry”), rather than simply “liking” a post. This change may alter how participants perceive support, as approximately 12% of participants reported friends provided support by liking posts. With this increased specificity, friends may choose a reaction, rather than posting a text response. Additional research is necessary to determine how these changes impact the objective support given and perceptions of support received.

Increasing mental health literacy may be another way to improve how friends provide support and individuals request/receive mental health-related support. Increasing mental health literacy has been shown to have a positive effect on reducing mental health stigma and may encourage friends to respond to depressive posts in more helpful ways [40-42]. In addition, Armstrong and Young [40] found that college students in their study preferred that mental health information be delivered via the internet, public service announcements, and the media. Providing health education and positive social support through social media or other online platforms may be one approach to support individuals experiencing depression.

### Limitations

This study was limited by several factors. First, the study was cross-sectional and used a convenience sample. The sample size was small (n=33), which also limits the generalizability of the findings. Thus, the understanding of an individual’s perceptions of support seeking and receiving may not generalize to all Facebook users, but are more likely to be generalizable to individuals with depression who have posted about or sought support related to depression, particularly when triangulated with findings from other research studies. In addition, the individuals who responded to the open-ended question about depression-related posts and were thus included in this analysis had higher depression scores than other individuals in the overall multisite study. Thus, perceptions of online support seeking and receipt are likely most generalizable to an individual with more significant depression symptoms. Finally, interactions on Facebook may not be generalizable to other social media platforms. Some young people are likely to disclose sensitive information on social media platforms other than Facebook, due to privacy concerns and concerns about parental presence on Facebook [26,43].

### Conclusions

This study explored how individuals perceived support seeking and their perceptions of the responses. The findings, when combined with those of prior studies, indicate that individuals may perceive their depression-related posts and the related responses differently from how others perceive them. As such, there may be a need to increase mental health literacy to help individuals, including both support seekers and support providers, communicate about mental health. Since stigma is often related to a lack of mental health literacy, enhancing mental health literacy may be an approach to reduce mental health stigma and increase support to individuals with depression symptoms.

## Appendix

Multimedia Appendix 1 Study questions about depression posts on Facebook.

## Abbreviations

IRB: institutional review board
PHQ-9: Patient Health Questionnaire-9

## References

[1] Gallagher RP (2007). National Survey of Counseling Center Directors, 2006 (Monograph Series No. 8P).

[2] American College Health Association (2009). American College Health Association: National College Health Assessment II: Reference Group Data Report Fall 2008.

[3] Zivin K, Eisenberg D, Gollust SE, Golberstein E (2009). Persistence of mental health problems and needs in a college student population. J Affect Disord.

[4] Hunt J, Eisenberg D (2010). Mental health problems and help-seeking behavior among college students. J Adolesc Health.

[5] Kessler RC, Foster CL, Saunders WB, Stang PE (1995). Social consequences of psychiatric disorders, I: educational attainment. Am J Psychiatry.

[6] Bramesfeld A, Platt L, Schwartz FW (2006). Possibilities for intervention in adolescents’ and young adults’ depression from a public health perspective. Health Policy.

[7] Deas D, Brown ES (2006). Adolescent substance abuse and psychiatric comorbidities. J Clin Psychiatry.

[8] Rao U (2006). Links between depression and substance abuse in adolescents: neurobiological mechanisms. Am J Prev Med.

[9] Garlow SJ, Rosenberg J, Moore JD, Haas AP, Koestner B, Hendin H, Nemeroff CB (2008). Depression, desperation, and suicidal ideation in college students: results from the American Foundation for Suicide Prevention College Screening Project at Emory University. Depress Anxiety.

[10] Rao U, Chen LA (2009). Characteristics, correlates, and outcomes of childhood and adolescent depressive disorders. Dialogues Clin Neurosci.

[11] Eisenberg D, Golberstein E, Gollust E (2007). Help-seeking and access to mental health care in a university student population. Med Care.

[12] (2018). Pew Research Center.

[13] (2018). Pew Research Center.

[14] Zhang R (2017). The stress-buffering effect of self-disclosure on Facebook: an examination of stressful life events, social support, and mental health among college students. Comput Hum Behav.

[15] Shensa A, Sidani JE, Escobar-Viera CG, Chu KH, Bowman ND, Knight JM, Primack BA (2018). Real-life closeness of social media contacts and depressive symptoms among university students. J Am Coll Health.

[16] Saunders JF, Eaton AA (2018). Snaps, selfies, and shares: how three popular social media platforms contribute to the sociocultural model of disordered eating among young women. Cyberpsycho Behav Soc Netw.

[17] Vogel EA, Rose JP, Okdie BM, Eckles K, Franz B (2015). Who compares and despairs? The effect of social comparison orientation on social media use and its outcomes. Pers Individ Differ.

[18] Frison E, Eggermont S (2015). Exploring the relationships between different types of Facebook use, perceived online social support, and adolescents’ depressed mood. Soc Sci Comput Rev.

[19] Murrieta J, Frye CC, Sun L, Ly LG, Cochancela CS, Eikey EV, Chowdhury G, McLeod J, Gillet V, Willett P (2018). Depression: findings from a literature review of 10 years of social media and depression research. Transforming Digital Worlds. iConference 2018. Lecture Notes in Computer Science.

[20] Moreno MA, Jelenchick LA, Egan KG, Cox E, Young H, Gannon KE, Becker T (2011). Feeling bad on Facebook: depression disclosures by college students on a social networking site. Depress Anxiety.

[21] Gowen K, Deschaine M, Gruttadara D, Markey D (2012). Young adults with mental health conditions and social networking websites: seeking tools to build community. Psychiatr Rehabil J.

[22] Radovic A, Gmelin T, Stein BD, Miller E (2017). Depressed adolescents’ positive and negative use of social media. J Adolesc.

[23] Egan KG, Koff RN, Moreno MA (2013). College students’ responses to mental health status updates on Facebook. Issues Ment Health Nurs.

[24] Park J, Lee DS, Shablack H, Verduyn P, Deldin P, Ybarra O, Jonides J, Kross E (2016). When perceptions defy reality: the relationships between depression and actual and perceived Facebook social support. J Affect Disord.

[25] Cavazos-Rehg PA, Krauss MJ, Sowles S, Connolly S, Rosas C, Bharadwaj M, Bierut LJ (2016). A content analysis of depression-related tweets. Comput Human Behav.

[26] Kroenke K, Spitzer RL, Williams JB (2001). The PHQ-9: validity of a brief depression severity measure. J Gen Intern Med.

[27] Richardson LP, McCauley E, Grossman DC, McCarty CA, Richards J, Russo JE, Rockhill C, Katon W (2010). Evaluation of the patient health questionnaire-9 item for detecting major depression among adolescents. Pediatrics.

[28] Allgaier A-K, Pietsch K, Frühe B, Sigl-Glöckner J, Schulte-Körne G (2012). Screening for depression in adolescents: validity of the patient health questionnaire in pediatric care. Depress Anxiety.

[29] Cash SJ, Bridge J (2012). Meeting them where they are: an exploration of technology use and help-seeking behaviors among adolescents and young adults.

[30] Krippendorf K (2004). Content Analysis: An Introduction to its Methodology.

[31] Miles M, Huberman M (1994). Qualitative Data Analysis: An Expanded Sourcebook.

[32] Weber RP (1990). Basic Content Analysis.

[33] American Psychiatric Association (2000). Diagnostic and Statistical Manual of Mental Disorders..

[34] Moreno MA, Jelenchick LA, Kota R (2013). Exploring depression symptom references on Facebook among college freshman: a mixed methods approach. Open J Depress.

[35] Holahan CJ, Moos RH, Holahan CK, Cronkite RC, Randall PK (2004). Unipolar depression, life context vulnerabilities, and drinking to cope. J Consult Clin Psychol.

[36] Rookm KS (1984). The negative side of social interaction: impact on psychological well-being. J Pers Soc Psychol.

[37] Davila J, Hershenberg R, Feinstein JA, Gorman K, Bhatia V, Starr LR (2012). Frequency and quality of social networking among young adults: associations with depressive symptoms, rumination, and corumination. Psychol Pop Media Cult.

[38] Krumpal I (2013). Determinants of social desirability bias in sensitive surveys: a literature review. Qual Quant.

[39] (2019). Facebook.

[40] Armstrong LL, Young K (2015). Mind the gap: person-centred delivery of mental health information to post-secondary students. Psychosoc Interven.

[41] (2007). Canadian Alliance on Mental Illness and Mental Health.

[42] Keys S, Cash S (2012). Reachout.com report to California Mental Health Association.

[43] Mullen C, Hamilton NF (2016). Adolescents' response to parental Facebook friend requests: he comparative influence of privacy management, parent-child relational quality, attitude and peer influence. Comput Human Behav.

