# Supplementary material for: What College Students Post About Depression on Facebook and the Support They Perceive: Content Analysis
Source: JMIR Form Res. 2020 Jul 17;4(7):e13650. doi: 10.2196/13650 (PMC7395250; doi:10.2196/13650)
Supplement: Multimedia Appendix 1 [file formative_v4i7e13650_app1.docx]

# Multimedia Appendix

**Now we’d like to transition to some questions about depression.  In some of our previous research studies, we have noted that some college students use social media, such as Facebook and Twitter, to display feelings and emotions related to depression.**

1.  Have you ever reached out on Facebook for help when depressed?

1b. If yes, can you tell us what type of post you made?

1c. If yes, how did your friends respond?
